# Measurement of Striatal Dopamine Release Induced by Neuropsychological Stimulation in Positron Emission Tomography With Dual Injections of [^11^C]Raclopride

**DOI:** 10.3389/fpsyt.2022.811136

**Published:** 2022-07-12

**Authors:** Yoko Ikoma, Yasuyuki Kimura, Makiko Yamada, Takayuki Obata, Tetsuya Suhara, Hiroshi Ito

**Affiliations:** ^1^Department of Molecular Imaging and Theranostics, Institute for Quantum Medical Science, National Institutes for Quantum Science and Technology, Chiba, Japan; ^2^Department of Functional Brain Imaging, Institute for Quantum Medical Science, National Institutes for Quantum Science and Technology, Chiba, Japan; ^3^Department of Clinical and Experimental Neuroimaging, Center for Development of Advanced Medicine for Dementia, National Center for Geriatrics and Gerontology, Obu, Japan; ^4^Department of Radiology and Nuclear Medicine, Fukushima Medical University, Fukushima, Japan

**Keywords:** positron emission tomography, [^11^C]raclopride, dopamine release, binding potential, dual-bolus injection, cognitive task

## Abstract

**Objectives:**

Positron emission tomography (PET) with [^11^C]raclopride has been applied to measure changes in the concentration of endogenous dopamine induced by pharmacological challenge or neuropsychological stimulation by evaluating the binding potential (BP) between the baseline and activated state. Recently, to reliably estimate BP in the activated state, a new approach with dual-bolus injections in a single PET scan was developed. In this study, we investigated the feasibility of applying this dual-bolus injection approach to measure changes in endogenous dopamine levels induced by cognitive tasks in humans.

**Methods:**

First, the reproducibility of BP estimation using the dual-bolus injection approach was evaluated using PET scans without stimulation in nine healthy volunteers. A 90-min scan was performed with bolus injections of [^11^C]raclopride administered at the beginning of the scan and 45 min after the first injection. BPs in the striatum for the first injection (BP_1_) and second injection (BP_2_) were estimated using an extended simplified reference tissue model, and the mean absolute difference (MAD) between the two BPs was calculated. The MAD was also compared with the conventional bolus-plus-continuous infusion approach. Next, PET studies with a cognitive reinforcement learning task were performed on 10 healthy volunteers using the dual-bolus injection approach. The BP_1_ at baseline and BP_2_ at the activated state were estimated, and the reduction in BP was evaluated.

**Results:**

In the PET scans without stimulation, the dual-bolus injection approach showed a smaller MAD (<2%) between BP_1_ and BP_2_ than the bolus-plus-continuous infusion approach, demonstrating good reproducibility of this approach. In the PET scans with the cognitive task performance, the reduction in BP was not observed in the striatum by either approach, showing that the changes in dopamine level induced by the cognitive tasks performed in this study were not sufficient to be detected by PET.

**Conclusion:**

Our results indicate that the cognitive task-induced changes in dopamine-related systems may be complex and difficult to measure accurately using PET scans. However, the proposed dual-bolus injection approach provided reliable BP estimates with high reproducibility, suggesting that it has the potential to improve the accuracy of PET scans for measuring changes in dopamine concentrations.

## Introduction

[^11^C]raclopride is an antagonist of dopamine D_2_ receptors, and positron emission tomography (PET) with [^11^C]raclopride has been widely used to image the binding of striatal dopamine D_2_ receptors in living humans (1–3). In PET dynamic acquisition, the binding potential (BP), which represents the density and apparent affinity of the receptors, can be quantified by analyzing the time course of the radioactivity concentration using a compartment model including compartments for free and specifically bound tracer in tissue (4–6) or some simplified models (7, 8), and it has been used to report changes in the density of dopamine D_2_ receptors in neurodegenerative or psychiatric disease (9–11).

Subsequently, PET studies with [^11^C]raclopride have been applied as a new approach for investigating changes in the concentration of endogenous dopamine due to pharmacological challenges or neuropsychological stimulations. The administered [^11^C]raclopride competes with endogenous dopamine for binding to the dopamine D_2_ receptors, and an increase in the concentration of endogenous dopamine is supposed to result in a decrease in the binding of administered [^11^C]raclopride, as measured by PET. Using this competition paradigm, some PET studies measured the BP before and after the stimulations and reported that the BP decreased after stimulation, such as an amphetamine challenge and behavioral or cognitive tasks (12–20).

To measure the changes in BP from a single PET scan, an injection protocol with a bolus-plus-continuous infusion has been applied (14, 15, 21). In these studies, stimulation was imposed during the continuous infusion, and BPs at the baseline and activated states were measured as the tissue-to-plasma concentration ratio at equilibrium using time frames before and after the stimulation. However, it is often difficult to design a protocol to attain equilibrium within the time frames of the baseline and activated states (22), and an unstable equilibrium may cause systematic errors in the BP estimates (19). On the other hand, two independent PET scans with a bolus injection for the baseline and activated states require a long study period and the physiological condition is often difficult to maintain. Therefore, shortening the scan time and improving the administration protocol and quantification method may reduce the burden on subjects and improve the reliability of the estimates.

Recently, a new approach for measuring changes in BP using a single PET scan with multiple bolus injections of [^11^C]raclopride has been proposed (23, 24). This approach provides the BP at the baseline and the activated state from data for the first and second injections by the extended simplified reference tissue model, and demonstrated that changes in BP after the second injection were detected in monkey studies when the binding conditions changed due to an increase in the administered mass of [^11^C]raclopride (23). However, this approach has not been applied to human studies investigating dopamine release caused by neuropsychological stimulations.

In this study, we evaluated the feasibility of applying this approach with dual-bolus injections of [^11^C]raclopride to detect changes in endogenous dopamine levels in human PET studies. First, the reproducibility of the BP estimates was evaluated in PET studies without stimulation. Second, changes in BP after stimulation were investigated in PET studies with cognitive tasks, and the results were compared with those of the conventional bolus-plus-continuous infusion approach.

## Materials and Methods

### Theory of Estimating Changes in Binding Potential With Dual-Bolus Injection

**FIGURE 1:**
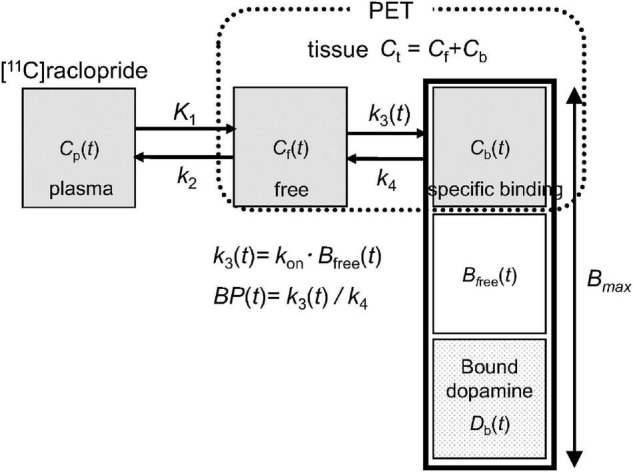
Extended compartment model including competition between administered [^11^C]raclopride and endogenous dopamine proposed in Endres et al. (21). Total receptor density (*B*_max_) is the sum of the free receptors (*B*_free_) and receptors bound by raclopride (*C*_b_) and dopamine (*D*_b_). A model parameter *k*_3_(*t*), representing the rate constant from the free compartment (*C*_*f*_) to the specific binding compartment (*C*_b_), depends on the association rate constant of [^11^C]raclopride (*k*_*on*_) and density of available receptors (*B*_free_).

In PET receptor imaging, the binding of administered radioligand to receptors can be quantified using a two-tissue compartment model, as shown in Figure 1 (21). In the baseline state, the number of receptors bound by [^11^C]raclopride (*C*_b_) and dopamine (*D*_b_) was considerably smaller than the total number of receptors (*B*_max_). On the other hand, in the activated state, *D*_b_ becomes larger and the number of available receptors (*B*_free_) decreases, resulting in a decrease in the binding of the administered [^11^C]raclopride obtained by the *BP* (= *k*_3_/*k*_4_). Therefore, changes in endogenous dopamine levels can be measured by comparing the BP between the baseline and activated states.

In the dual-bolus injection approach, bolus injection of [^11^C]raclopride is performed twice in a single session of PET scanning. The first injection of [^11^C]raclopride is performed at the time of the scan initiation, and the *BP* of the baseline is estimated by the usual simplified reference tissue model with a reference region instead of an arterial input function (7). Then, the second injection is administered immediately before the change in the binding conditions without waiting for decaying radioactivity of the first injection. The *BP* of the activated state is estimated using data after the second injection by the multiple-injection simplified reference tissue model (MI-SRTM), including the remaining radioactivity of the first injection, as follows (23, 24):


$$C_{\mathrm{t1}}\,\left(t\right)=R_{1}\,C_{r}\,\left(t\right)+\left(k_{2}-\frac{R_{1}\,k_{2}}{1+B\,P}\right)\cdot \exp\left(-\frac{k_{2}}{1+B\,P}\,t\right)\,⊗C_{r}\,\left(t\right)$$

$$C_{\mathrm{t2}}\,\left(t\right)=R_{1}^{\prime }\,C_{r}\,\left(t\right)+\left(k_{2}^{\prime }-\frac{R_{1}^{\prime }\,k_{2}^{\prime }}{1+B\,P^{\prime }}\right)\cdot \exp\left(-\frac{k_{2}^{\prime }}{1+B\,P^{\prime }}\,t\right)$$

(1) $$\;\,⊗C_{r}\,\left(t\right)+\left(C_{\mathrm{t0}}-R_{1}^{\prime }\,C_{\mathrm{r0}}\right)\cdot \exp\left(-\frac{k_{2}^{\prime }}{1+B\,P^{\prime }}\,t\right)$$

where *C*_*t*1_ and *C*_t2_ are the radioactivity concentrations in the target region for the first and second injections, respectively, *C*_*r*_ is the radioactivity concentration in the reference region, *t* is the time from the first or second injection, and *C*_*t*0_ and *C*_r0_ are the radioactivity concentrations of the target and reference regions at the time of the second injection, respectively. *K*_1_ and *k*_2_ are the rate constants of the compartment model in the target region shown in Figure 1, and *R*_1_ is the ratio of *K*_1_ between the target and reference regions. The mathematical symbol ⊗ represents the convolution operation.

### Subjects

PET scans were performed for 19 healthy men (mean age ± *SD*, 23.2 ± 2.8 years). Nine subjects were under resting condition until the end of the scan without stimulation to evaluate the reproducibility of the *BP*. Meanwhile, the other 10 subjects were subjected to a cognitive task during the scan to induce dopamine release to investigate if changes in *BP* could be detectable. All subjects underwent two PET scans on separate days; one was the scan by the proposed dual-bolus injection protocol (dual-bolus method) and the other was the scan by the conventional bolus-plus-continuous infusion protocol (bolus-infusion method). All subjects were free of somatic, neurological, or psychiatric disorders. This study was approved by the Ethics and Radiation Safety Committee of the National Institute of Radiological Sciences. Written informed consent was obtained from all participants before inclusion in the study. The study was registered with University Hospital Medical Information Network Clinical Trials Registry (UMIN000008221, UMIN000013753).

### Positron Emission Tomography Procedure

PET studies with [^11^C]raclopride were performed using an Eminence SET-3000GCT/X (Shimadzu Corp., Kyoto, Japan). This scanner provides 99 planes with a 26-cm axial field of view, and the intrinsic spatial resolution was 3.4 mm in-plane and 5.0 mm full-width at half maximum (FWHM) axially (25). A 10-min transmission scan was acquired before an emission scan using a ^137^Cs line source for subsequent attenuation correction. [^11^C]raclopride was administered by the dual-bolus method or bolus-infusion method, and emission data were acquired in three-dimensional mode. The list-mode data were rebound to sinograms and reconstructed by filtered back-projection using a Gaussian filter (cutoff frequency, 0.3 cycle/pixel). The reconstructed in-plane resolution was 7.5 mm in FWHM and the voxel size of the reconstructed images was 2.0 × 2.0 × 2.6 mm. The head movement of each frame was corrected by frame-by-frame image realignment with attenuation correction using a resliced μ-map, as reported previously (26).

T1-weighted magnetic resonance (MR) images were acquired with a GE 3.0-T Excite system (slice thickness 1.0 mm; matrix, 256 × 256; field of view, 25 × 25 cm).

#### Protocol of Dual-Bolus Method

**FIGURE 2:**
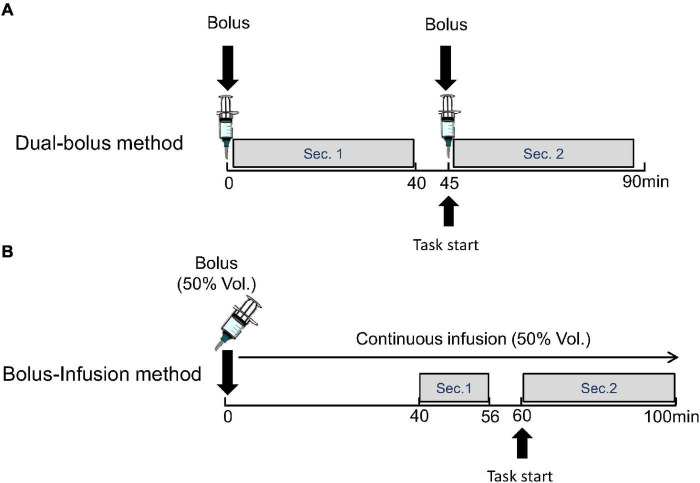
Protocol of proposed dual-bolus injection method **(A)** and conventional bolus-plus-continuous infusion method **(B)**. Binding potentials were estimated using frames before the stimulation (Sec. 1) and after the stimulation (Sec. 2).

In the dual-bolus method, [^11^C]raclopride was taken in two syringes for the first and second injections. A bolus of 217.7 ± 10.3 MBq [^11^C]raclopride was administered at the time of scan initiation, and a bolus of 195.2 ± 13.2 MBq [^11^C]raclopride was administered subsequently. The interval between the first and second injection was 45.2 ± 0.27 min. The specific activity of the first and second injection was 224.8 ± 62.7 GBq/μmol and 48.3 ± 13.4 GBq/μmol, respectively, at the time of each injection. The emission data were acquired in three-dimensional mode over 90 min. The list-mode data were rebound to sinograms as 69 time-frames of 20-s × 12 frame, 60-s × 16 frame, 240-s × 6 frame, and 60-s × 1 frame after the first injection, 20-s × 12 frame, 60-s × 16 frame, and 240-s × 6 frame after the second injection. In the scans with the stimulations, the preparation of the cognitive task started at 40-min and the actual performance started at approximately 45-min after the scan initiation and soon after the second injection (Figure 2A).

The BP of the first section (*BP*_1_) as the baseline, and the BP of the second section (*BP*_2_) as the activated state were estimated using time-activity curves (TACs) for 40-min data after the first and second injections by the MI-SRTM (Eq. 1), with the cerebellum as the reference region.

#### Protocol of Bolus-Infusion Method

In the bolus-infusion method, [^11^C]raclopride was set in the syringe pump, and half of this (261.4 ± 17.5 MBq) was administered by a bolus injection at the time of scan initiation, which was followed by a continuous infusion of the remaining [^11^C]raclopride over 100 min. The specific activity was 171.7 ± 54.0 GBq/μmol at the start of the scan. Note that the ratio of injection volume between the bolus and continuous infusion was designed by a computer simulation so that the TAC would be flat after 38-min.

The emission data were acquired in three-dimensional mode over 100 min. The list-mode data were rebound to sinograms as 48 time-frames of 20-s × 12 frame, 60-s × 16 frame, and 240-s × 20 frames. In the scans with the stimulation, the preparation of the cognitive task started at 56-min and the actual performance started at 60-min after beginning the scan (Figure 2B).

The BP was calculated from the TAC of the target and reference regions on the assumption that the radioactivity concentration in the reference region reflects the concentration of unbound [^11^C]raclopride in the target region (15) as follows:


(2) $$B\,P=\frac{A\,U\,C_{\text{tar}}-A\,U\,C_{\text{ref}}}{A\,U\,C_{\text{ref}}}\;$$

where *AUC*_tar_ and *AUC*_ref_ are the areas under the TAC of the target and reference regions, respectively. The BP of the two sections (*BP*_1_ and *BP*_2_), which are the baseline and activated states, were calculated from the areas under the TAC between 40 and 56 min and between 60 and 100 min, respectively.

#### Cognitive Task

In this study, the participants performed cognitive reinforcement learning with the probabilistic selection task reported previously (27) as a neuropsychological stimulation during the scan. Briefly, six symbols resembling Sanskrit alphabet were prepared, and three different symbol pairs were presented on the display in random order. The participants selected the desirable symbol by the selection switch to hit the correct answer while viewing the symbols on the display, and then feedback was turned to indicate whether it was correct or incorrect. However, this feedback was probabilistic, and each pair had a different probability of returning correct feedback. Over the course of training, the participants learned to choose symbols with a higher probability of correct feedback.

#### Preprocessing of Data Analysis

**FIGURE 3:**
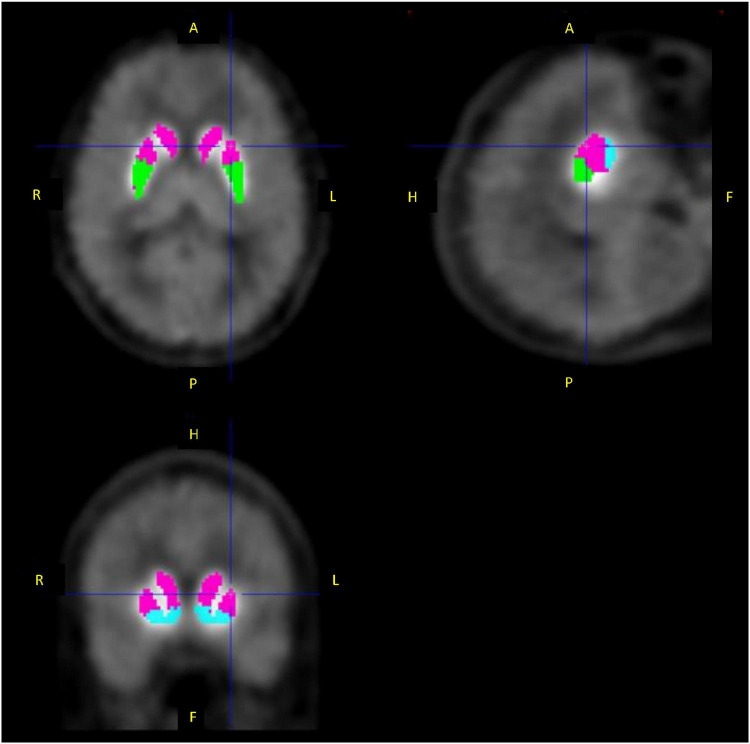
Regions of interest for the limbic striatum (blue), executive striatum (pink), and sensorimotor striatum (green).

PET dynamic images were summed for all frames and coregistered to the individual MR images. Then, individual MR images were normalized to the template of MR T1 image, of which the matrix size was 91 × 109 × 91 and voxel size was 2.0 × 2.0 × 2.0 mm, by DARTEL in SPM12 (Wellcome Centre for Human Neuroimaging, UCL Queen Square Institute of Neurology, London, United Kingdom), and PET images were also normalized to the template using the same transformation parameters. Regions of interest (ROIs) were drawn manually on the template MR image for the striatum subdivisions, including the limbic striatum (495 voxels), executive striatum (939 voxels), sensorimotor striatum (416 voxels), and whole striatum merged these subdivisions (Figure 3). The cerebellum was also delineated as the reference region, where the specific binding was negligible (6, 28, 29). The respective TACs were then extracted from the normalized dynamic PET images.

All data analyses were performed using the PMOD software (PMOD Technologies, Zurich, Switzerland) and MATLAB (Mathworks, Natick, MA, United States).

### Evaluation of Reproducibility in the Binding Potential

The reproducibility of BP estimates was evaluated by comparing *BP*_1_ and *BP*_2_ in the PET scans without stimulation, assuming the case where no binding change was induced during the scan. From each TAC of the striatum ROIs, *BP*_1_ and *BP*_2_ were obtained as mentioned above, and the reproducibility of BP estimates was evaluated by the mean absolute difference (MAD) and intraclass correlation coefficient (ICC) (30) as follows:


(3) $$MAD\left(\%\right)=\frac{\left|B\,P_{1}-B\,P_{2}\right|}{\left(B\,P_{1}+B\,P_{2}\right)/2}\cdot 100$$

(4) $$I\,C\,C=\frac{M\,S\,B-M\,S\,W}{M\,S\,B+M\,S\,W}$$

where MSB is the mean sum of squares between subjects, and MSW is the mean sum of squares within subjects. The MAD and ICC obtained using the dual-bolus method were compared with those obtained using the bolus-infusion method.

### Evaluation of Changes in the Binding Potential Induced by Cognitive Task

In the PET scans with the stimulation of cognitive tasks, *BP*_1_ at the baseline and *BP*_2_ at the activated state were estimated as mentioned above for each TAC of the striatum ROIs, and the change in BP was evaluated as follows:


(5) $$△BP\left(\%\right)=\frac{B\,P_{2}-B\,P_{1}}{B\,P_{1}}\cdot 100$$

The Δ*BP*s obtained using the dual-bolus method were compared with those obtained using the bolus-infusion method. A statistical comparison between *BP*_1_ and *BP*_2_ was performed using paired-samples *t*-tests with a significance level of 0.05.

## Results

### Evaluation of Reproducibility in the Binding Potential

In PET images without stimulation, a high accumulation of [^11^C]raclopride was observed in the striatum. In the whole striatum, *BP*_1_ and *BP*_2_ by the dual-bolus injection method was 2.50 ± 0.10 and 2.48 ± 0.11, respectively (Table 1). There was no significant difference in BP estimates between the dual-bolus and bolus-infusion methods. The MAD of the whole striatum was 1.10 ± 0.66 and the ICC was 0.959 in the dual-bolus method, which was superior to those in the bolus-infusion method. The maximum MAD among the nine subjects was 2.3% in the dual-bolus injection method. Meanwhile, in the bolus-infusion method, three of nine subjects showed a higher MAD (>2.7%) and the maximum MAD was 2.9%. With respect to each striatal subdivision, the MAD in the dual-bolus method was 1.14 ± 0.62 in the limbic striatum, 1.32 ± 0.75 in the executive striatum, and 1.68 ± 1.44 in the sensorimotor striatum. These MAD values were smaller than those measured in the bolus-infusion method in all striatal subdivisions.

**TABLE 1:** Changes in binding potential for PET studies without stimulation.

	Dual-bolus method				Bolus-infusion method			
		
	BP_1_	BP_2_	MAD (%)	ICC	BP_1_	BP_2_	MAD (%)	ICC
	mean (*SD*)	mean (*SD*)	mean (*SD*)		mean (*SD*)	mean (*SD*)	mean (*SD*)	
Limbic striatum	2.22 (0.13)	2.21 (0.13)	1.14 (0.62)	0.974	2.28 (0.11)	2.27 (0.12)	1.57 (1.12)	0.927
Executive striatum	2.57 (0.11)	2.54 (0.13)	1.32 (0.75)	0.951	2.59 (0.13)	2.57 (0.13)	1.48 (1.23)	0.934
Sensorimotor striatum	2.69 (0.14)	2.67 (0.15)	1.68 (1.44)	0.922	2.70 (0.14)	2.68 (0.15)	1.90 (1.47)	0.905
Whole striatum	2.50 (0.10)	2.48 (0.11)	1.10 (0.66)	0.959	2.53 (0.11)	2.52 (0.11)	1.54 (1.10)	0.912

### Evaluation of Changes in the Binding Potential Induced by Cognitive Task

**FIGURE 4:**
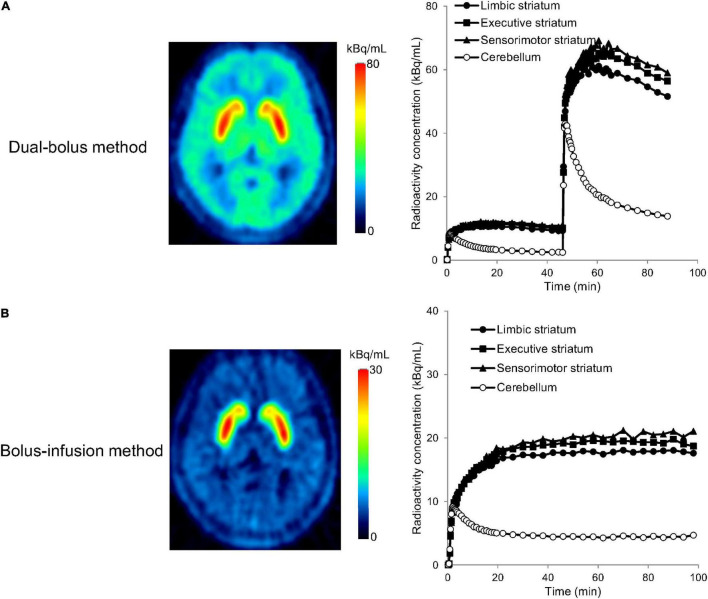
Positron emission tomography (PET) images and time-activity curves of the limbic striatum, executive striatum, sensorimotor striatum, and cerebellum for the dual-bolus method **(A)** and bolus-infusion method **(B)** in [^11^C]raclopride-PET studies with cognitive tasks. PET images show the averaged radioactivity concentration for the second section after the stimulation (45–89 min for the dual-bolus method, 60–100 min for the bolus-infusion method). Time-activity curves were decay corrected to the time of the scan start.

A typical example of the TACs for the striatal subdivisions and cerebellum in the PET studies with the stimulation of cognitive tasks is shown in Figure 4. In the whole striatum, Δ*BP* was –0.35 ± 1.53 (range, –2.22 to 2.29) in the dual-bolus method, and –0.31 ± 2.90 (range, –4.32 to 5.87) in the bolus-infusion method (Table 2). Despite the average *BP*_2_ value decreasing, no significant difference between *BP*_1_ and *BP*_2_ was observed using either method. Although the average Δ*BP* value was almost the same between the two methods, the variation of Δ*BP* in the dual-bolus method was considerably smaller than that of the bolus-infusion method. With respect to each striatal subdivision, Δ*BP* of the dual-bolus method and bolus-infusion method were 0.87 ± 2.13 (range, –2.18 to 3.84) and –0.31 ± 3.34 (range, –4.74 to 7.38), respectively, in the limbic striatum, –0.48 ± 1.43 (range, –2.52 to 2.37) and –0.53 ± 2.67 (range, –4.06 to 4.97), respectively, in the executive striatum, and –1.39 ± 2.15 (range, –5.12 to 1.73) and 0.19 ± 3.29 (range, –4.40 to 6.25), respectively, in the sensorimotor striatum. The reduction in *BP*_2_ was the largest in the sensorimotor striatum of the dual-bolus method; however, there was no significant difference between *BP*_1_ and *BP*_2_ in any of the striatal subdivisions in either method. The variation of Δ*BP* in the dual-bolus method was smaller than that in the bolus-infusion method in all striatal subdivisions.

**TABLE 2:** Changes in binding potential for PET studies with cognitive task performance.

	Dual-bolus method				Bolus-infusion method			
		
	BP_1_	BP_2_	*P-value	Δ BP (%)	BP_1_	BP_2_	*P-value	Δ BP (%)
	mean (*SD*)	mean (*SD*)		mean (*SD*)	mean (*SD*)	mean (*SD*)		mean (*SD*)
Limbic striatum	2.27 (0.27)	2.30 (0.30)	0.18	0.87 (2.13)	2.40 (0.39)	2.38 (0.38)	0.68	–0.31 (3.34)
Executive striatum	2.55 (0.14)	2.54 (0.15)	0.34	–0.48 (1.43)	2.61 (0.34)	2.60 (0.34)	0.50	–0.53 (2.67)
Sensorimotor striatum	2.75 (0.16)	2.72 (0.17)	0.078	–1.39 (2.15)	2.82 (0.39)	2.82 (0.40)	0.88	0.19 (3.29)
Whole striatum	2.52 (0.13)	2.51 (0.15)	0.52	–0.35 (1.53)	2.60 (0.34)	2.59 (0.33)	0.70	–0.31 (2.90)

*P-value of the paired-sample t-test between BP_1_ and BP_2_ of each method.

## Discussion

### Detection of Changes in Binding Potential by the Dual-Bolus Injection Method

In PET studies with [^11^C]raclopride, the measurement of changes in the BP due to pharmacological or neuropsychological stimulations has been attempted using various approaches, such as a scan with bolus-plus-continuous infusion, two separate PET scans with a bolus injection, and a single session of PET scan with extended simplified reference tissue model including time-dependent parameters (12–16, 18–20). In the conventional bolus-plus-continuous infusion approach, the BP can be estimated by the ratio of radioactivity concentration between the striatum and cerebellum at equilibrium, and the calculation process is easy and stable. However, it is often difficult to maintain an equilibrium state during scanning. In our studies using the bolus-infusion method, we designed the injection protocol by a computer simulation using the average kinetic rate constants of [^11^C]raclopride for humans so that the time-activity curve became flat at 38 min after scan initiation. However, even in the PET scans with the cognitive tasks, the area under the TAC of the whole striatum between 60 and 100 min was larger than that between 40 and 56 min in nine of ten subjects, and three of them increased by more than 5%. This suggests that the TAC did not reach equilibrium and continued to increase during the scan. Therefore, the reliability of BP estimates may depend on the equilibrium condition among individuals and on the time range used for BP estimation. Subsequently, an extended simplified reference tissue model was developed to quantify the BP reduction in the non-equilibrium state from a 90-min scan with bolus-plus-continuous infusion (19). Nevertheless, continuous infusion requires the equipment to provide [^11^C]raclopride constantly. Moreover, the signal-to-noise ratio deteriorates due to the radioactivity decaying in the delayed frames used for *BP*_2_ estimation. This results in lower reliability of *BP*_2_ estimates compared to that of *BP*_1_ estimates.

The dual-bolus method overcame these problems by performing a second bolus injection at almost the same time as the start of change in the binding condition and estimates BP in the activated state by a compartmental analysis using data after the second injection. In this method, almost the same radioactivity concentration of [^11^C]raclopride is administered 45 min after the first injection, and the injection protocol is simple. Quantification analysis is also simple and is the same as the usual simplified reference tissue model, except that it requires a constant parameter of residual radioactivity at the time of the second injection, as shown in Eq. 1. Furthermore, the second bolus injection increased the PET count in delayed frames, resulting in images with high signal-to-noise ratio even in the frames for *BP*_2_ estimation. Therefore, the dual-bolus method is expected to provide *BP*_2_ estimates as reliably as *BP*_1_ estimates. In our studies for the evaluation of reproducibility, the MAD of the dual-bolus method was smaller than that of the bolus-infusion method and was also small enough compared with those obtained from two separate PET scans with a bolus injection of [^11^C]raclopride reported previously (30), demonstrating good reproducibility of this dual-bolus method.

### Changes in Binding Potential Induced by the Cognitive Task Performance

As a next step, we applied this dual-bolus method to measure the changes in BP induced by the cognitive task. It was expected that BP would decrease after cognitive task performance because of the increase in dopamine concentration. However, no significant reduction in BP was observed with either the dual-bolus or bolus-infusion methods. Phasic bursts of dopamine cell firing during positive reinforcement have been reported in animals (31, 32) and a similar elevation of dopamine levels has been suggested in humans (33, 34). Nevertheless, our results suggest that the amount of dopamine released by the cognitive reinforcement learning task performed in this study was not sufficient to induce changes in BP. The previous monkey study with amphetamine challenge and microdialysis analysis reported that the ratio of percent dopamine increase to percent striatal binding reduction for amphetamine (0.2 mg/kg) was 44:1 and demonstrated that relatively small binding changes reflect large changes in dopamine level (14). This means that a fivefold increase in dopamine concentration induces a 10% decrease in BP. Unlike dopamine elevation by pharmacological challenge, the amount of dopamine release as a result of cognitive performance is supposed to be smaller. However, some studies with behavioral or cognitive tasks have reported a reduction in striatal BP (16, 18); for example, about 13% BP reduction in the ventral striatum was observed during a video game (16), demonstrating the possibility of measuring the dopamine release induced by neuropsychological stimulation as well as the pharmacological challenge using [^11^C]raclopride-PET. On the other hand, our study did not detect any changes in BP by both injection methods, which suggests the difficulty of measuring the changes in BP due to psychological stimulation reproducibly by [^11^C]raclopride-PET. Unlike the pharmacological dopamine elevation, the cognitive task-induced changes in dopamine-related systems may be complex, thus hindering the prediction of the magnitude of BP changes before the PET measurement. Despite our attempts to investigate dopamine level changes using the cognitive reinforcement learning task, as previously reported (27), further investigations with different stimulations may be needed to fully assess the matter. Additionally, a stimulation protocol optimization may improve the dual-bolus method’s potential for stably evaluating the BP changes induced by cognitive tasks.

In the [^11^C]raclopride-PET scans with cognitive tasks, the reliability of parameter estimates is important to evaluate small changes in BP. Considering the results of PET studies with the cognitive task as shown in Table 2, the variation of *BP*_1_, *BP*_2_, and Δ*BP* among subjects in the dual-bolus method was considerably smaller than that in the bolus-infusion method, as well as that in the PET studies without the cognitive task. These results suggest that the stability of the estimated parameters originated from technical improvements of the dual-bolus method. Detection of BP change in the dual-bolus method has already been demonstrated in an animal study with a large mass of radiotracer administration (23). Therefore, this stability of parameter estimation in PET studies with the cognitive task is an advantage of the dual-bolus injection method.

### Potential Limitations of the Dual-Bolus Injection Method

The dual-bolus method can provide BP with high reproducibility and detect changes in the binding of [^11^C]raclopride, as reported in monkey studies (23). However, this method requires caution. First, as with the bolus-infusion method, the dual-bolus method employs the TAC of the cerebellum as the reference region instead of using the arterial input function. Strictly speaking, radioactivity concentration in the cerebellum is different from radioactivity concentration of unbound [^11^C]raclopride in the striatum shown as *C*_f_ in Figure 1, and this causes bias in BP estimated using the cerebellum TAC compared with that using the arterial input function. However, it is practically difficult to reliably obtain the arterial input function and estimate *k*_3_/*k*_4_ directly without the reference region. Although the BP estimates may cause some bias by using the reference region, the previous studies reported these values became smaller after changes in the dopamine level induced by amphetamine challenge in the bolus-infusion method (14, 15) or by an increase in molar amount of administered [^11^C]raclopride in the dual-bolus method (23). Furthermore, computer simulations based on the extended compartment model shown in Figure 1 demonstrated that the integral of dopamine pulse concentration was related to the magnitude of reduction in BP using the reference region (21, 23). These results suggest changes in the dopamine level can be detectable as changes in BP using the reference region. Second, the magnitude of BP reduction is affected by various factors other than stimulus-induced dopamine elevation, such as the mass and kinetics of the administered ligand. It has been reported that a large molar mass with low specific activity of administered [^11^C]raclopride causes a reduction in BP due to an increase in the number of bound receptors (23). In our study, BP reduction was not observed in PET scans without stimulation, and the MAD was smaller than that of the bolus-infusion method, suggesting that the specific activity is sufficiently high to avoid BP reduction. Finally, in the simplified reference tissue model, BP is estimated based on the assumption that it is constant during the data frames after the bolus injection. Therefore, when the dopamine concentration changes with time after the stimulation, the magnitude of BP reduction is considerably affected by the timing of dopamine release and the shape of dopamine concentration changes over time (35). These dopamine releases may differ depending on the kind of stimulation. Although it is difficult to measure them directly in humans, more optimization for the design of cognitive task performance may improve the sensitivity to BP reduction in the dual-bolus method.

In summary, we performed PET studies with dual-bolus injections of [^11^C]raclopride to measure BP reduction induced by cognitive tasks. Although the binding of [^11^C]raclopride did not change after the cognitive reinforcement learning task, this method could provide the BP of two sections assuming the baseline and activated state with high reproducibility from a single session of PET scanning. Our results suggest that the dual-bolus injection method has the potential to improve the accuracy with which we are able to measure changes in dopamine levels. Additionally, it may be beneficial for future research regarding the investigation of changes in dopamine concentrations induced by various pharmacological or neuropsychological stimuli.

## Data Availability Statement

The raw data supporting the conclusions of this article will be made available by the authors, without undue reservation.

## Ethics Statement

The studies involving human participants were reviewed and approved by the Ethics and Radiation Safety Committee of National Institute of Radiological Sciences. The patients/participants provided their written informed consent to participate in this study.

## Author Contributions

YI: conceptualization, methodology, data analysis, and writing-original draft. YK: conceptualization, methodology, PET data collection, and writing-review, and editing. MY: conceptualization, design and implementation of cognitive tasks, and writing-review, and editing. TO and TS: discussion on results and writing-review, and editing. HI: conceptualization, methodology, PET data collection, supervision, and writing-review, and editing. All authors have approved the manuscript.

## Conflict of Interest

The authors declare that the research was conducted in the absence of any commercial or financial relationships that could be construed as a potential conflict of interest.

## Publisher’s Note

All claims expressed in this article are solely those of the authors and do not necessarily represent those of their affiliated organizations, or those of the publisher, the editors and the reviewers. Any product that may be evaluated in this article, or claim that may be made by its manufacturer, is not guaranteed or endorsed by the publisher.
